# Effect of Bone Morphogenetic Protein-2 (BMP-2)/Hydroxyapatite/In Situ-Formed Hyaluronan Hydrogel Composites on Bone Formation in a Murine Model of Posterolateral Lumbar Fusion

**DOI:** 10.7759/cureus.25509

**Published:** 2022-05-30

**Authors:** Akiyoshi Kuroda, Wataru Saito, Gen Inoue, Masayuki Miyagi, Shintaro Shoji, Hiroyuki Sekiguchi, Masashi Takaso, Kentaro Uchida

**Affiliations:** 1 Department of Orthopaedic Surgery, Kitasato University School of Medicine, Sagamihara, JPN; 2 Department of Orthopaedics, Medical Sciences Research Institute, Shonan University, Chigasaki, JPN

**Keywords:** hyaluronan, hydroxyapatite, posterolateral lumbar fusion, in situ-formed hydrogel, bone morphogenetic protein-2

## Abstract

Several types of calcium phosphate (CaP) biomaterial carriers have been designed to deliver bone morphogenetic protein-2 (BMP-2) to augment spinal fusion in spinal surgery. Here, we evaluated an in situ-formed hydrogel (IFH) constructed from hyaluronan (IFH-HA) combined with a BMP2/hydroxyapatite (HAP) composite in bone formation in a murine model of posterolateral lumbar fusion (PLF). HAP was submerged in HA-tyramine (TA) polymer solution containing horseradish peroxidase (HRP) and 2 µg BMP-2 (BMP2/HA-TA/HRP solution). H_2_O_2_ was added to initiate the curing reaction (BMP-2/IFH-HA). phosphate-buffered saline (PBS) was added to the BMP2/HA-TA/HRP solution (BMP-2/HA-TA) instead of H_2_O_2_ to evaluate the effectiveness of the curing reaction. HAP immersed in PBS was used as a control. PLF model mice were randomly assigned to receive one these composites (n = 10 each).

X-ray images were taken to assess the bone fusion, and microcomputed tomography analysis was conducted to examine new bone formation at the graft site four weeks following surgery. No evidence of fusion was observed four weeks after surgery in the Control or BMP2/HA-TA group. In contrast, the BMP2/IFH-HA group exhibited newly formed bone between the transverse processes and bone union in coronal sections. Relative to the Control and BMP2/HA-TA groups, the BMP2/IFH-HA group showed significantly greater bone volume. The BMP2/IFH-HA group also showed significantly elevated bone mineral content relative to the BMP2/HA-TA group. A composite comprising BMP2/HAP and IFH-HA, thus, enhanced the new bone formation in a murine model of PLF, suggesting its promise for augmenting spinal fusion.

## Introduction

Posterolateral lumbar fusion (PLF) is a common procedure for spinal fusion. For solid fusion, bone stock needs to be added to facilitate fusion of the bone from two isolated sites. Bone stock typically contains autologous bone, allogeneic bone, and artificial bone. Although autologous bone can be obtained locally, there is a risk of complications and pain at the site of bone collection.

In the orthopedic field, calcium phosphate (CaP) biomaterials such as synthetic hydroxyapatite (HAP) and beta-tricalcium phosphate (β-TCP) are often adopted to facilitate bone formation [1]. The composition and microstructure of CaP closely resemble those of native bone tissue to provide favorable surroundings for bone formation [2,3]. However, due to the limited osteoinductive activity of CaP, the material can be disadvantageous for bone formation at graft sites, which have an inactive osseous environment [4,5].

Bone morphogenetic protein-2 (BMP-2) is an osteoinductive protein that facilitates the recruitment and differentiation of mesenchymal progenitor cells (MPCs) into the osteoblastic lineage, which go on to form a bony matrix. Approved by the United States Food and Drug Administration, BMP-2 is widely used as a bone treatment [6,7]. Delivery of BMP-2 on an absorbable collagen sponge (ACS) is an approved technique for certain interbody fusion procedures. Additionally, a number of different CaP carriers have been reported to deliver BMP-2 for the purpose of actively facilitating bone formation in PLF [8,9]. However, the initial burst release pattern observed using these CaP carriers has the potential to decrease bone formation and induce adverse effects in the clinic [10,11].

To overcome this problem, we focused on in situ-formed hydrogels (IFHs) constructed from natural polysaccharides, including collagen, gelation, dextran, pullulan, and hyaluronan (HA) [12-17]. IFHs cure via an oxidative coupling reaction between hydrogen peroxide (H_2_O_2_) and horseradish peroxidase (HRP) in an aqueous solution. In our previous studies, we reported that IFH constructed from HA (IFH-HA) enabled a sustained release of BMP-2 at a fracture and bone defect site [18,19]. However, the effect of combining BMP-2 with CaP remains unclear. In this article, we introduced BMP-2 into HAP using IFH-HA and examined the effect of a BMP-2/IFH-HA/HAP composite on bone formation in a murine model of PLF.

## Materials and methods

Synthesis of graft materials

**Figure 1:**
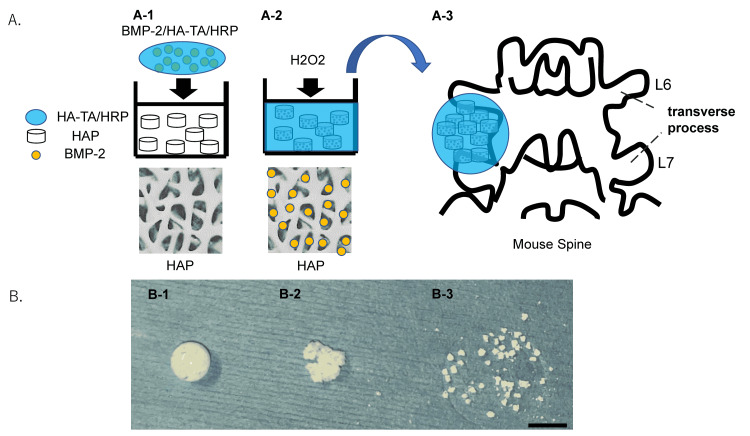
Creation of BMP2/hydroxyapatite/in situ-formed hyaluronan hydrogel composites (A and B) Scheme showing how bone morphogenetic protein-2 (BMP-2)/hydroxyapatite (HAP)/in situ-formed hyaluronan hydrogel (IFH-HA) composites were formed. A-1: Hyaluronan-tyramine (HA-TA) polymer solution containing horseradish peroxidase (HRP) and BMP-2 (BMP-2/HA-TA/HRP) was added to HAP. A-2: To initiate the curing reaction, H_2_O_2_ was added to the HAP in BMP2/HA-TA/HRP solution (BMP2/IFH-HA). A-3: BMP2/HAP/IFH-HA composite was grafted between the L6 and L7 transverse processes. B-1: 4 mM H_2_O_2_ was added to the HAP in BMP2/HA-TA/HRP solution to initiate curing. B-2: PBS was added to the BMP2/HA-TA/HRP solution instead of H_2_O_2_ to evaluate the effectiveness of the curing reaction. B-3: HAP in PBS was used as a control.

We generated the hyaluronan-tyramine (HA-TA) conjugate based on details described elsewhere [18]. To synthesize IFH-HA, the HA-TA polymer was crosslinked in the presence of the catalyzing enzyme HRP (FUJIFILM Wako Pure Chemical Corporation, Richmond, VA) and H_2_O_2_ in 10 mM phosphate-buffered saline (PBS; pH 7.4). HAP (10 mg) was submerged in 2% HA-TA polymer solution with 0.8 units/mL HRP and 2 µg BMP-2 (PeproTech Inc., Rocky Hill, NJ) (Figure 1, Panel A-1). To initiate the curing reaction, 4 mM H_2_O_2_ was added to the HAP in BMP2/HA-TA/HRP solution (BMP-2/IFH-HA, Figure 1, Panels A-2, B-1). PBS was added to the BMP2/HA-TA/HRP solution instead of H_2_O_2_ to evaluate the effectiveness of the curing reaction (BMP-2/HA-TA, Figure 1, Panel B-2). HAP in PBS was used as a control (Figure 1, Panel B-3).

Mouse model of PLF

The animals were handled and subjected to surgeries according to the guidelines of the Animal Ethics Committee of Kitasato University (Permission number: 2020-090). Thirty C57BL/6J mice (aged 10 weeks, male; Charles River Laboratories Japan, Inc., Yokohama, Japan) chosen for this study were provided standard laboratory chow (CRF-1, Oriental Yeast, Tokyo, Japan) and housed under controlled temperature (25 ± 1°C), humidity (60 ± 5%), and light conditions (12-hour light/dark cycle).

To anesthetize mice, diethyl ether was injected intramuscularly into the upper limbs together with a 1:10 dilution of a mixture containing one part midazolam, three parts Domitor, and one part Vetorphale at 0.15 ml per animal. To establish the PLF model, a cut was made in the skin along the midline and then in the paramedian fascia right of L6-L7 aseptically to reveal the lumbar posterolateral spine. Using blunt dissection to divide the dorsal paraspinal muscles allowed the laminae and costal processes of L6-L7 to become exposed. BMP2/IFH-HA, BMP2/HA-TA, or PBS was then positioned in the right lateral space linking the L6 and L7 costal processes.

Microcomputed tomographic (μCT) analysis

Four weeks after the PLF surgery, all mice were euthanized using an overdose of CO_2_ inhalation, and the lumbar spine and pelvis were removed along with the surrounding muscle. The extracted tissue was fixed in 4% paraformaldehyde (Nacalai Tesque Inc., Kyoto, Japan) at 4°C for 48 hours. Subsequently, μCT images were taken with a micro-focus x-ray and CT system (inspeXio SMX-90CT Plus; Shimadzu, Tokyo, Japan) at voxel size, 20 μm/pixel; matrix size, 1024 × 1024; acceleration voltage, 90 kV; and current, 110 mA. Images were taken in the coronal plane from the L6 to L7 transverse process to assess the posterolateral bone mass. Further, images were taken along the axial plane from the L6 to L7 transverse process and at the midline between these vertebrae, and the mean cross-sectional area of newly formed bone was measured on the surface of the vertebrae with three-dimensional (3D) image analysis software (Tri-3D-Bon; Ratoc System Engineering, Tokyo, Japan) (Figure 1, Panel B). To evaluate the volume of new bone, we constructed a HAP calibration curve using the data generated from phantom images obtained using various densities of HAP (Ratoc System Engineering, Tokyo, Japan). To assess the bone mineral content in each sample, densities measured using the microcomputed tomography (μCT) images were compared with the HAP calibration curve. A threshold of 300 to 800 mg/cm^3^ was used to indicate the presence of new bone as grafted HAP has a density greater than 800 mg/cm^3^.

Histology

Following μCT analysis, the tissues were embedded in methyl methacrylate, sectioned at 4 μm thickness, and reacted with Masson’s trichrome stain to visualize the formation of new bone between the costal processes 6 and 7.

Statistical analysis

Statistical comparisons were conducted in Statistical Package for the Social Sciences (SPSS) version 25.0 (IBM Corp., Armonk, NY). We used ANOVA and then Bonferroni's posthoc comparisons test to compare groups. p < 0.05 suggested a statistical significance.

## Results

Evaluation of new periosteal bone formation from x-ray and µCT images

**Figure 2:**
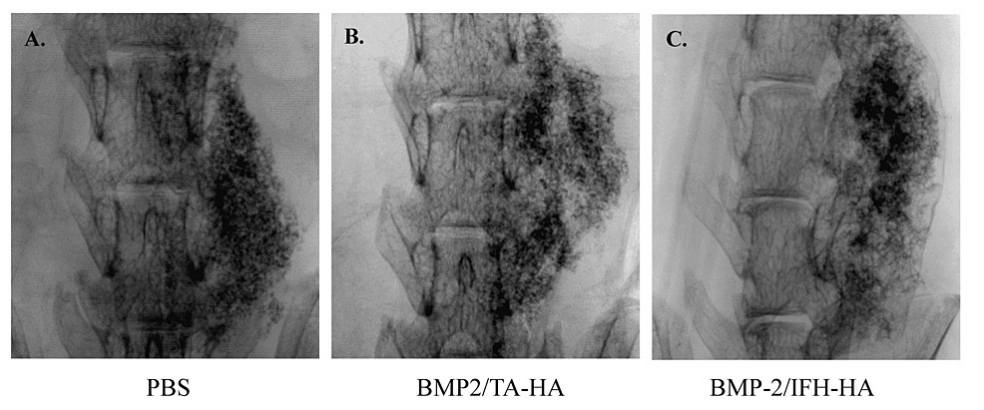
Soft x-ray images of the region surrounding L6 and L7 four weeks following surgery (A) PBS, (B) BMP2/HA-TA, and (C) BMP2/IFH-HA groups. PBS: Phosphate-buffered saline; BMP2: Bone morphogenetic protein-2; HA: Hyaluronan; TA: Tyramine; IFH: In situ-formed hydrogel.

**Figure 3:**
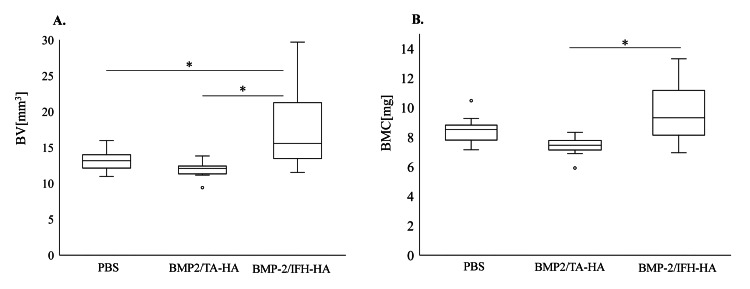
Bone volume and bone mineral content of graft sites four weeks following surgery Microcomputed tomography images were analyzed for new bone formation four weeks following surgery in the PBS, BMP2/HA-TA, and BMP2/IFH-HA groups. (A) Bone volume (BV) and (B) bone mineral content (BMC). n = 10. *p < 0.05. PBS: Phosphate-buffered saline; BMP2: Bone morphogenetic protein-2; HA: Hyaluronan; TA: Tyramine; IFH: In situ-formed hydrogel.

No evidence of bone fusion was observed four weeks after surgery in the Control group and BMP2/HA-TA group (Figure 2, Panels A, B; fusion rate, Control group, 0/10; BMP2/HA-TA group, 0/10). In contrast, the BMP2/IFH-HA group showed newly formed bone between costal processes 6 and 7 and bone union in coronal sections at the same time point (Figure 2, Panel C; fusion rate, 10/10; p < 0.001). Meanwhile, all groups showed new bone formation on the surface of the laminae and spinal processes. Bone volume was significantly elevated in the BMP2/IFH-HA group relative to the PBS and BMP2/HA-TA groups (p = 0.028 and p = 0.001, respectively, Figure 3, Panel A). Further, the BMP2/IFH-HA group had elevated bone mineral content relative to the BMP2/HA-TA group (p = 0.003, Figure 3, Panel B).

Histology

**Figure 4:**
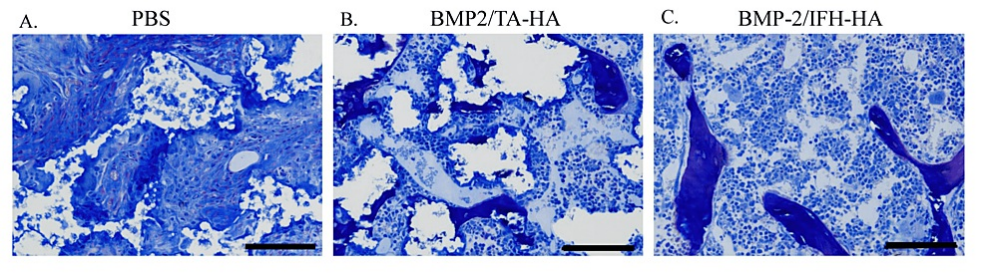
Histological images showing new bone formation four weeks following surgery Masson’s trichrome staining was used to reveal new bone formation at the graft site. (A) PBS, (B) BMP2/HA-TA, and (C) BMP2/IFH-HA groups. Scale bars indicate 100 μm. PBS: Phosphate-buffered saline; BMP2: Bone morphogenetic protein-2; HA: Hyaluronan; TA: Tyramine; IFH: In situ-formed hydrogel.

Consistent with our µCT results, histological examination revealed abundant bone formation in the BMP-2/IFH-HA group and little newly formed bone in the BMP-2/HA-TA and Control groups (Figure 4).

## Discussion

Delivery of BMP-2 on an ACS is an approved technique for certain interbody fusion procedures. Off-label use of BMP-2/ACS wrapped in synthetic bone consisting of β-TCP and HAP has demonstrated superior efficacy to autologous cancellous bone grafts in PLF [20]. Here, we compared the usefulness of a BMP2/HAP/IFH-HA composite with a BMP-2/HAP/TA-HA composite for stimulating bone formation in a murine model of PLF. Our findings suggest that an IFH-HA/HAP/BMP-2 composite may be a new treatment method for augmenting spinal fusion.

Activation of BMP2 signaling in the early phase of the bone regeneration process can cause inflammation and activation of MPCs, and plays a pivotal role in the osteoblastic differentiation of MPCs [21-24]. This suggests that extended and continued release of BMP2 could be pertinent for speeding up bone formation at graft sites. In a previous study, an IFH-HA material that was capable of continually releasing platelet-derived growth factors over 14 days enhanced the proliferation of MPCs in vitro [25]. Likewise, in our previous study, IFH-HA was able to continually release BMP2 for 14 days in vitro [18]. Together, the present study and previous studies suggest that BMP2/HAP/IFH-HA composites may exhibit high bone induction potential owing to their ability to continually release BMP-2.

However, a limitation of our study is that findings from rodent models may not necessarily be translatable to the clinic owing to different responses to BMP-2 in humans [26]. Additional studies in large animal models are needed to validate our results.

## Conclusions

We evaluated an in situ-formed hydrogel constructed from hyaluronan combined with a BMP2/hydroxyapatite composite in the bone formation in a murine model of PLF. A composite comprising BMP2/HAP and IFH-HA enhanced new bone formation in a murine model of PLF, suggesting its promise for augmenting spinal fusion.
